# Conformational changes influence clogging behavior of micrometer-sized microgels in idealized multiple constrictions

**DOI:** 10.1038/s41598-019-45791-y

**Published:** 2019-06-25

**Authors:** Izabella Bouhid de Aguiar, Martine Meireles, Antoine Bouchoux, Karin Schroën

**Affiliations:** 1 Laboratory of Food Process Engineering, Wageningen University, Wageningen, The Netherlands; 2 Laboratoire de Génie Chimique, Université de Toulouse, CNRS, INPT, UPS, Toulouse, France; 3 Laboratoire d’Ingénierie des Systèmes Biologiques et des Procédés, CNRS, INRA, INSAT, Université de Toulouse, 31400 Toulouse, France

**Keywords:** Chemical engineering, Soft materials

## Abstract

Clogging of porous media by soft particles has become a subject of extensive research in the last years and the understanding of the clogging mechanisms is of great importance for process optimization. The rise in the utilization of microfluidic devices brought the possibility to simulate membrane filtration and perform *in situ* observations of the pore clogging mechanisms with the aid of high speed cameras. In this work, we use microfluidic devices composed by an array of parallel channels to observe the clogging behavior of micrometer sized microgels. It is important to note that the microgels are larger than the pores/constrictions. We quantify the clog propensity in relation to the clogging position and particle size and find that the majority of the microgels clog at the first constriction independently of particle size and constriction entrance angle. We also quantify the variations in shape and volume (2D projection) of the microgels in relation to particle size and constriction entrance angle. We find that the degree of deformation increases with particle size and is dependent of constriction entrance angle, whereas, changes in volume do not depend on entrance angle.

## Introduction

The observation of soft particles going through constrictions has become a subject of extensive research in the last years^1–3^. Depending on the properties and size of the particle relative to the pore size, various mechanisms occur. Micrometer sized soft particles reduce their size or deform to go through constrictions that are smaller than their diameter, whereas hard particles would not be able to do so. Small soft particles moving through larger pores would not need to deswell or deform, leading to colloidal interactions playing a more prominent role^4,5^.

For hard particles, clogging can happen through sieving effects, bridging or agglomeration, depending on the size (distribution) of the particles, and their interactions^6^. Since these particles are not able to modify their conformation, the clogging propensity is determined by process conditions and the ratio of channel to particle (agglomerate) dimension^6^. When using a suspension of colloidal soft particles, clogging can happen through agglomeration or the formation of arches, as was the case for hard particles; but the pore size is no longer the strict gate keeper for particle permeation^7,8^. Soft particles larger than constrictions may be pushed all the way through;^1^ whereas the largest particles have highest propensity to get stuck in the constrictions and clog the channels^9^.

Depending on particle size and constriction geometry, soft particles can use two mechanisms to accommodate the pressure drop generated by the clog they generate: deformation and deswelling^10,11^. Soft particles deform when they assume a different shape to adjust to the surrounding environment. Deformation can also be observed in emulsion droplets^2,12^, however, unlike emulsion droplets, soft particles such as microgels are also able to accommodate some of the external pressure by deswelling. Deswelling happens when particles expel solvent, therewith effectively decreasing their volume. Studies have already shown that mostly both deformation and deswelling of constricted soft particles occur simultaneously. The challenge that we face is to understand how these modifications occur, and use that knowledge in the design of processes where these effects occur (such as membrane filtration).

Membrane filtration is an operation widely used to process soft particles as for example in waste water treatment^13,14^, and dairy filtration^15,16^. The biggest challenges are cake layer formation and membrane fouling^17,18^, in both cases, membranes lose part of their permeability and consequently the filtration process loses efficiency^19^. Understanding the behavior of soft particles during membrane filtration is therefore essential.

Microfiltration is a membrane filtration process based on size exclusion that has pressure as a driving force. The size exclusion concept says that what is larger than the size of the pore will be retained while what is smaller will go through^19,20^. In a process where soft particles are present, the concept of size exclusion should be used with care since, as soft particles are able to modify their conformation under pressure making the size exclusion concept not very straightforward, since soft particles can be pushed into and even through the membrane pores. For soft particles, the pore size is only relevant if it can be linked to the effective size of the particles under process conditions.

Many direct and indirect methods have been reported in the literature to observe and describe the filtration behavior of soft particles. Some indirect methods include the quantification of soft particles in the permeate, and modeling of process parameters^12,21^. Direct observation methods have also been described and mostly include the use of microfluidic devices coupled with optical microscopes and high-speed cameras^3,22^. Microfluidic devices are flexible in their design and allow for endless variation in conformation. In our work, the microfluidic devices generally contain constricted channels and soft particles flow through them. We chose to work with microgels as model particles due to their ease of fabrication and tunable properties. The novelty of our work is to observe individual (deswelling and deformation) and collective particle behavior (clog propensity, system selectivity) and correlate these parameters.

In this work, we use microfluidic devices to observe the clogging behavior of microgels larger than the constrictions. We quantify the clog propensity in relation to the clogging position and particle size and find that the majority of the microgels clog at the first constriction independent of particle size and constriction entrance angle. We also quantify the variations in shape and volume (2D projection) of the microgels in relation to particle size and constriction entrance angle. We find that the degree of deformation increases with particle size and is dependent on constriction entrance angle, whereas, changes in volume are not dependent on entrance angle.

## Material and Methods

### Microgel synthesis and characterization

Microgels were synthesized and characterized as described in previous work^23^. Polyacrylamide (pAAm) microgels are used as model particles for the channel clogging experiments. The microgels were produced via the emulsion templating technique as described in previous work^24^. For the preparation of the microgels, we start by mixing 100 ml kerosene with 1% of the surfactant polyglycerol polyricinoleate (PGPR90). In a separate container we prepare the monomer solution with 10 ml of water, 2.5 g of acrylamide, 50 mg of potassium persulfate (KPS) and 25 mg of N,N′-methylenebisacrylamide (BIS) as the crosslinker at 1%wt as compared to the total monomer content. We use drops of 0.1 M sodium hydroxide solution to set the monomer solution pH to 8.5. The monomer solution is added to the content of the round bottom flask and the aqueous phase is emulsified into the oil phase under high shear (13000 rpm) with a rotor-stator mixer (IKA ultraturrax) for three minutes. We then close the round bottom flask with a rubber septum and bubble the emulsion with nitrogen for 20 minutes to remove oxygen. We subsequently place the round bottom flask on a stirring plate on ice and we inject 1 ml N,N,N′,N′-tetramethylethylenediamine (TEMED) to trigger the polymerization. The reaction time is 2–3 hours. After, we precipitate the microgels in cold methanol and the microgels are cleaned by repeated centrifugation and resuspension steps, first in methanol to remove excess kerosene and surfactant, and finally in water. The microgel suspension is stored at 4 °C. The microgels are micrometer sized and present a polydisperse size distribution. The diameter of the microgels range from 3 µm to 50 µm with a D^2,3^ of 10 µm. Size distribution was measured by laser diffraction (Malvern Mastersizer 3000). The microgels were used suspended in water at ~0.1%vol.

### Microfluidics

**Figure 1:**
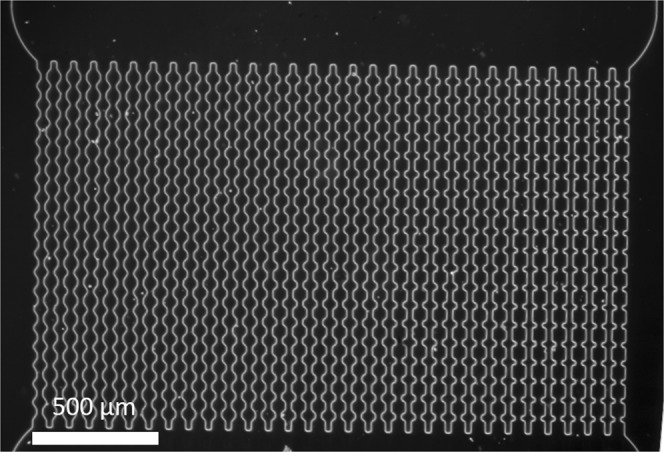
Optical microscopy image of the array of channels that compose the microfluid device at 2.5x magnification. There are 30 parallel channels with five different entrance angles. From right to left: 0°, 20°, 35°, 45°, 55°.

**Figure 2:**
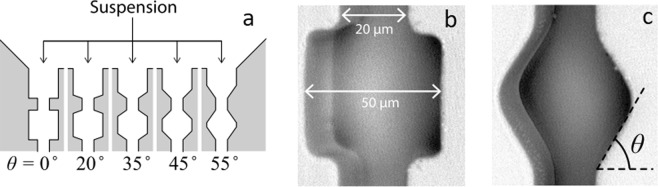
(**a**) Schematic representation of the channels and their different entrance angles, (**b**) internal dimensions of the constrictions, depth of the channels is 40 µm, (**c**) constriction representing the location where the angles are measured. Reproduced with permission from Nature (van de Laar *et al*.^25^).

Microfluidic experiments were performed as described in previous work^23^. The microfluidic devices we use to simulate membrane filtration are composed of a main channel with an inlet and an outlet. An array of smaller parallel channels is connected to the main channels to simulate the pores of a membrane. The channels have 5 different entrance angles (6 channels for each angle) varying from 0° to 55° (Figs 1,2)^25^. The microfluidic devices were produced by soft lithography and coupled to an optical microscope equipped with a high-speed camera to allow *in situ* observation of the filtration process. The devices were connected via Teflon tubing to a pressure controller (Elveflow OB1-MK3). The filtration experiments were conducted at 100 mbar and observed at relatively low magnification (2.5x) until all the channels were clogged. We consider the system completely clogged when there is at least one particle permanently stuck in every channels in the time frame observed (average of 10 minutes). After system clogging was complete, we increased the magnification to 20x, zooming in on a single particle stuck in a pore and gradually increased the pressure up to 800 mbar to force the particle to go through the constriction. With this experiment we expect to be able to observe particle’s change in conformation and/or volume when going through a constriction. In previous work, we found a total pressure drop of 10% across the totality of the channel^8^.

### Image analysis

Image analysis was performed as described in previous work^23^. Self-written matlab scripts are used to analyze the images; for the clogging experiments, we determine the average clog constriction position, so how deep each channel clogs. For experiments focusing on the single particle behavior, we select images from the sequence obtained with a high-speed camera: typically showing the particles in the middle of the constriction, on the verge of being pushed all the way through. We use these images to determine the degree of deformation of the particles as previously described^24^. Briefly, we rewrote the sphericity equation in terms of the area and the perimeter of the microgel in a 2D image which leads to the following equation to calculate this sphericity parameter *Ψ*:

1 $$\psi =\frac{2\sqrt{\pi {N}_{a}}}{{N}_{circ}}$$

where *N*_*a*_ is the number of pixels in the area of a microgel and *N*_*circ*_ is the number of pixels in the circumference of the microgel area.

## Results and Discussion

### Average clog position in the channels

**Figure 3:**
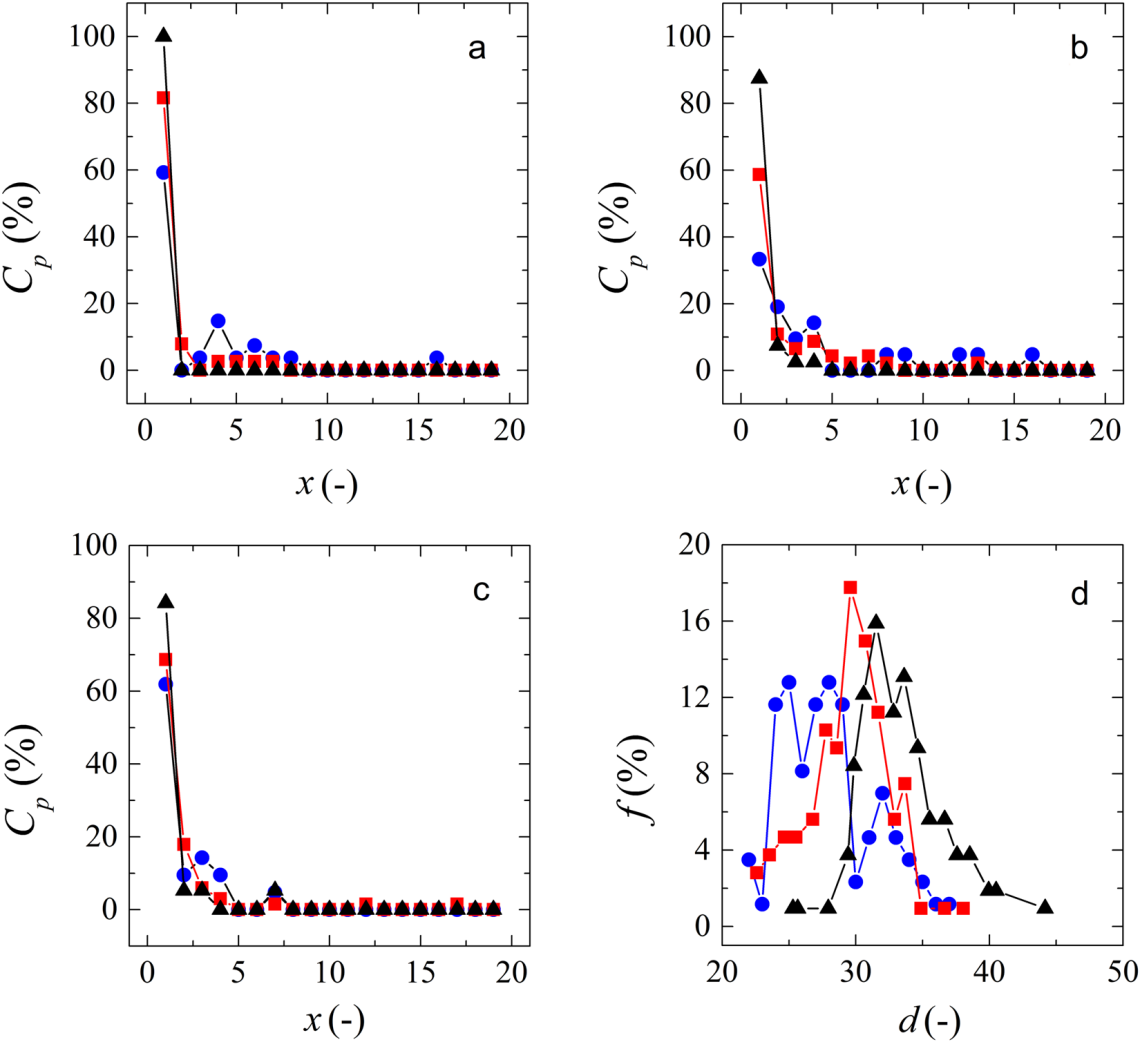
Typical relationship between clog propensity and clog position (how deep in the channel the clog stabilizes) within one experiment at (**a**) 50 mbar, (**b**) 100 mbar and (**c**) 150 mbar. Blue symbols represent particles between 25 and 30 µm. Red symbols represent particles between 31 and 35 µm and black symbols represent particles between 36 and 40 µm. (**d**) Size distribution of the microgels that clog the pores at 50 mbar (blue symbols), 100 mbar (red symbols) and 150 mbar (black symbols). Lines are to guide the eye.

In this study, we observe the behavior of individual particles at local scale through optical microscopy. During the clogging experiments, the channels of the microfluidic devices clog at different positions. To analyze whether this position is related to the particle size or to the applied pressure, we plot the clog propensity for each clog position for three different filtration pressures (Fig. 3a–c). Clog propensity is the percentage of clogs in a determined position in relation to the total amount of observed clogs in all channels. For a better understanding of the data, we classified the particles that clogged the pores according to their size in three different size ranges within the same experiment. From our observations it is clear that most of the channels clog at the first constriction, independently of particle size or applied pressure. There is a slight trend for smaller particles to clog at higher constriction number (deeper in the channel), which seems obvious since smaller particles are more likely to be pushed through a constriction, having to go through less shape modification (deswelling and deformation).

At 50 mbar applied pressure, we can see that clogging happens only in the first position for particles larger than 30 µm. For smaller particles, this happens within the first eight constrictions. As we increase the applied pressure to 100 mbar, the particles may be forced to go deeper into the channels before clogging it. Particles larger than 30 µm have a very high propensity to clog at the first constrictions and smaller particles have higher propensity to clog at deeper positions. As we increase the applied pressure to 150 mbar, we see that particles smaller than 25 µm do not clog the channels anymore (Fig. 3c,d). The pressure is high enough to promote the modification of their conformation and for that reason they can be pushed all the way through the channels. Slightly larger particles show a high propensity to clog at the beginning of the channels, as part of the natural sieving effect. Given the process conditions, these particles cannot modify their shape sufficiently to move through the constrictions, and thus clog the channels. The size distribution of the particles that clog the channels at different pressures can be seen in Fig. 3d. We can see that the system selectivity changes with the variation in pressure due to the changes in conformation of the microgels.

The channels of the microfluidic devices used in this work have a variety of entrance angles. In previous work^23^, we found that the entrance angles do not have an influence on overall clogging behavior of micrometer-sized particles that are smaller than the pore. Here we investigate larger particles in detail.

### *In situ* observation of clogging microgels

**Figure 4:**
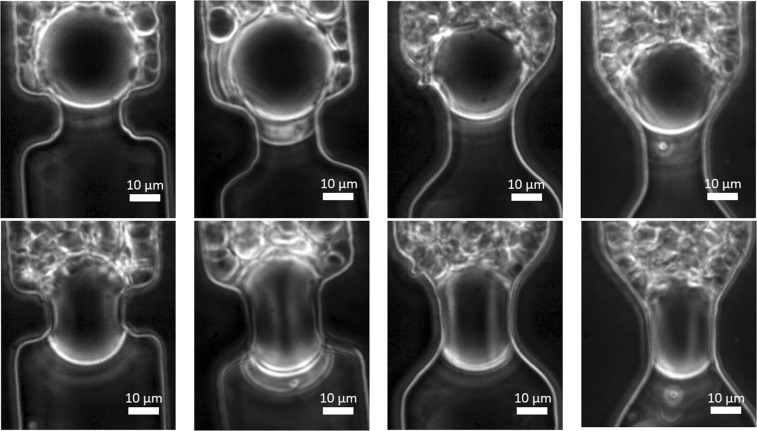
Optical microscopy (20x) images of microgels before constrictions (top) and in the middle of constrictions (bottom), for four different entrance angles going from left to right: 0, 20, 45 and 55°.

Whenever soft particles are forced through pores that are smaller than their diameter, the particles change their shape as illustrated in Fig. 4 that shows microgels before going through a constriction and during their passage through the constriction. It is easy to see that the microgels go through changes in shape (deformation); however, it is harder to quantify deformation and to analyze whether microgels also deswell (loose volume). For that reason, we use image analysis to determine the degree of deformation and deswelling of the particles, as shown in the next sections.

To determine the degree of deformation of the microgels before and during their passage though the pores (middle of the constriction), we determine the sphericity parameter of the microgels from the images (Equation 1).

For example, the sphericity of a perfect sphere is 1.00, the sphericity of an ellipse is 0.93, and the sphericity of a dumbbell is approximately 0.80. These are also the shapes we will use throughout the paper to describe the deformed microgels.

**Figure 5:**
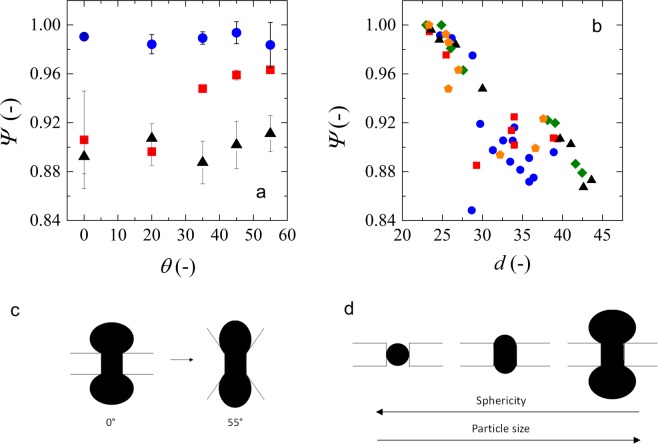
(**a**) Microgels sphericity (*Ψ*) variation with entrance angle (*θ*). Blue circles represent data obtained for microgels with diameters between 24 and 26 µm, red squares for diameters between 27 and 30 µm, and black triangles for diameters higher than 30 µm. (**b**) Sphericity as function of particle diameter. Blue circles represent entrance angles of 0°, red squares 20°, black triangles 35°, green diamonds 45° and orange pentagons 55°. (**c**) Scheme representing the difference in shape that microgels assume at different entrance angles. (**d**) Scheme representing the change in shape of at increasing microgel size.

In Fig. 5a, we plot the sphericity of microgels at different entrance angles for microgels with different sizes. The sphericity of microgels with diameters varying from 24 to 26 µm going through constrictions does not depend on the entrance angle of the channels and their values are very close to 1, which implies that these microgels do not deform much to go through the constrictions. This is not surprising since the constrictions have a size of 20 µm. As the microgels increase in size (diameters from 27 to 30 µm), they tend to have lower sphericity (Fig. 5b,d), which is more pronounced at low channel entrance angles (Fig. 5a). This means that the microgels are more deformed as a dumbbell when going through low entrance angle constrictions (Fig. 5c). On the other hand, at high entrance angles the microgels tend to assume a more elliptical shape, as reflected in their sphericity values.

**Figure 6:**
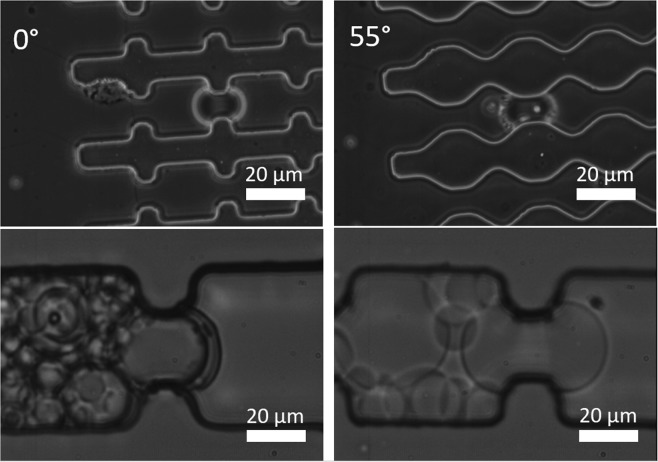
Top: Optical microscopy images (10x) showing microgels of similar size in the middle of constrictions of different entrance angles. Particles have diameters of 43 and 41 µm, respectively. Scale bars denote 50 µm. Bottom: Optical microscopy images (20x) of microgels with different sizes going through constrictions with the same entrance angle (0°). Particles have diameters of 27 and 43 µm, respectively.

For microgels with diameters larger than 30 µm, we observe an independence of microgel sphericity on entrance angle, and also find low sphericity values (Fig. 5a). This means that these microgels are so large that they have to deform to a dumbbell shape independently of the shape of the constriction. We also have illustrated a number of effects in Fig. 6, in which we show images of microgels of similar size going through low and high entrance angle constrictions (top part), and it is clear that both microgels deform into similar dumbbell shapes. When using different-sized microgels going through the same constriction as illustrated in the bottom images of Fig. 6, it is also clear that they assume different shapes. A video of a microgels going through a constrictions is available as a Supplementary file.

### Particle compression versus deformation

**Figure 7:**
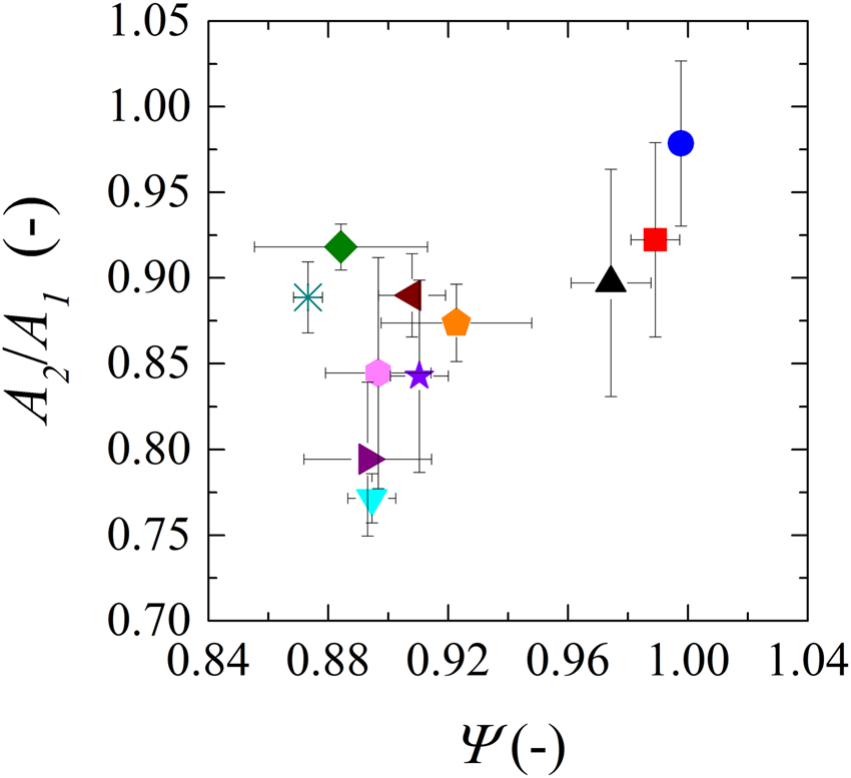
Comparison between deswelling (*A*_*2*_/*A*_*1*_) and deformation (*Ψ*) for microgels with diameters varying from 22 to 43 µm. Points represent binned data: 22–23 µm (blue circle), 24–25 µm (red square), 26–27 µm (black triangle), 28–29 µm (green diamond), 30–31 µm (orange pentagon), 32–33 µm (brown left arrow), 34–35 µm (purple right arrow), 36–37 µm (pink hexagon), 37–39 µm (lilac star), 40–41 µm (teal inverted triangle), 42–43 µm (turquoise asterisk).

To quantify compression/deswelling of the particle we use the ratio between the 2D projection area of the particle image in the middle of the constriction (A_2_) and the area of the particle before the constriction (A_1_). When presenting experimental results of particle deswelling, we will refer to the change in 2D projected area; which is used as an indicator for the change in 3D volume. We plot the change in area as function of the sphericity (Fig. 7) for particles of similar size ranges, this time for smaller size intervals as the ones used before (in Fig. 3). Small particles may lose a bit of area, but the sphericity is still very close to 1 since the particles hardly need to deform or deswell to pass the constriction. As the size of the microgels increases, both sphericity values and change in area decrease since larger microgels have to modify themselves more to go through the pores. As for the degree of each modification (deformation or deswelling) we see that deswelling plays an important role as does deformation. Larger particles obviously need to deform more to pass through the constriction, and possibly deswell to some extent. Both processes are time dependent, and since we binned all data irrespective of the applied pressure or entrance angle, it could be that some differences are due to the kinetics of these processes.

Also, large microgels (>40 μm) assume a dumbbell shape while going through the pores (Fig. 7). The dumbbell shape allows for a more localized compression, i.e. the deswelling occurs only at the part that is going through the constriction and as soon as that part is released, it swells back while another part is being compressed. In this way the microgels do not deswell as a whole but part by part and reswell quickly as soon as they come out of the constriction. For these reason, the variation in area for larger microgels as seen in Fig. 6 is again comparable to the ones found in smaller microgels. As mentioned previously, the angle and the applied pressure (kinetics) may have played a role here, but for now we consider this outside the scope of our analysis, and we limit ourselves to the overall behavior.

## Discussion

In previous work^24^, we investigated the behavior of microgel packings at different osmotic pressures, and found that microgels use both deswelling and deformation to accommodate pressure to different extents. The composition of the microgels changes with increasing pressure since they lose volume, becoming more concentrated which also influences their deswelling and deformation behavior. In our current observations (microgels under dynamic conditions) we see that this also holds. The microgels both deform and lose volume to accommodate applied forces as illustrated in Fig. 7, albeit that in our previous work the microgels were part of a packing and experienced an isotropic pressure. This is not the case in the current work in which microgels experience an anisotropic pressure while being forced through a constriction. In order to deform and deswell and go through a constriction, the microgels have the modify themselves in a much more dynamic way, deswelling and reswelling occur continuously and simultaneously. Also the extent of local particle modification was higher as for the isotropic pressure case, especially when the microgels are much bigger than the constriction aperture.

We observe that microgels clog individually due to the fact that they are soft and large. For this reason they are not likely to clog by forming arches or agglomerate (no colloidal effects)^2,6^, However, as they are soft they can also go through constrictions that are smaller than their diameter by changes in shape and volume^10^.

We observe that the majority of permanent clogs happen in the first constriction. Li et al^11^. measure the critical pressure at which microgels are forced through a constriction. They find that after going through a first constriction, a smaller critical pressure is necessary for the microgels to go through next constrictions if the particles do not reswell fast enough to their original shape and size. This would imply that once a particle passes the first constriction, it would pass all of them, but that is typically not what we find since clogging deep in the pores is also observed. Our microgels regain their initial shape and volume immediately after passing through a constriction. We measured the ratio between particle diameter before and after the constriction and obtained an average of 1.01. The critical pressure can also help us explain the change in system selectivity with pressure.

We also found that the change in shape of the microgels does depend on the channel entrance angle, whereas the variation in volume is not dependent on the channel entrance angle since the data is very scattered (see supplementary information). The same observation was made by Li *et al*.^10^. On small microgels the entrance angle does not have an influence on the shape of the microgels. This is because when they are inside the constrictions they are not in contact with the entrance of the channel but only with the internal dimensions of the constriction. For this reason, only the internal dimensions of the constriction will influence the deformation and deswelling of microgels. When the microgel is large, part of it will be inside the constriction and part outside. The part that enters the constriction loses volume and the rest of the microgel keeps their original properties. As soon as the part that was squeezed comes out of the constriction, it reswells. It is then the turn of the part that was still outside of the constriction to deswell. After passage the volume of the particle is similar as the entering particle, which is indicative of the very fast swelling/deswelling processes that occur.

In membrane filtration of soft particles, transmission through the pores can be observed, and is not always desired. Pan *et al*.^9^ synthesized fluorescently labelled microgels, and found microgels in the permeate when using microporous membranes with pores much smaller than the diameter of the particles. Understanding this transmission mechanism (also in relation to the pore size distribution) is important for process optimization when the presence of particles in the permeate is not desirable, or where a specific size separation is needed. In the current work, we showed that the pressure can also be used to influence the separation characteristics, but in a different was as customary in many separation processes in which it serves as the main driving force. The effect of different pore entrance angles can be seen as a link to the ‘membranes of the future’ that may have uniform pores, of which the actual geometry can be used to tune separation characteristics. Other example of areas that can benefit from our results are chromatography, injectable microgels for tissue engineering^26^ and studies on blood cell flow^27^.

## Conclusions

We used microfluidic channels with multiple constrictions as model membranes to observe microgel behavior, and found that microgels mostly clog the channels at the first constriction independently of constriction entrance angle, particle size and applied pressure. At higher pressures, slightly larger particles are needed to clog a constriction; the degree of deformation increases with particle size and is dependent on constriction entrance angle, whereas changes in volume are independent of entrance angle, which is indicative of very rapid swell/deswelling. Our findings are of great importance for especially membrane filtration in which the size of a particle is used as indication for retention. This however is obviously a very simplistic starting point for the soft particles investigated here since they are able to adjust their shape and size. Our results will become even more relevant when innovative membranes with uniform pores that are now used in our labs become available on large scale.

## Supplementary information

Particle compression

Microgel going through a constriction

## Data Availability

The datasets generated during and/or analyzed during the current study are available from the corresponding author on reasonable request.
