# Supplementary material for: Conformational changes influence clogging behavior of micrometer-sized microgels in idealized multiple constrictions
Source: Sci Rep. 2019 Jun 25;9:9241. doi: 10.1038/s41598-019-45791-y (PMC6592940; doi:10.1038/s41598-019-45791-y)
Supplement: Supplementary file 1 — Particle compression [file 41598_2019_45791_MOESM1_ESM.pdf]

Conformational changes influence clogging behavior of micrometer-sized microgels in idealized multiple constrictions.

Izabella Bouhid de Aguiar[1,2], Martine Meireles[2], Antoine Bouchoux[3], Karin Schroën[1]

[1] Laboratory of Food Process Engineering, Wageningen University, Wageningen, the Netherlands.
[2] Laboratoire de Génie Chimique, Université de Toulouse, CNRS, INPT, UPS, France
[3] Laboratoire d'Ingénierie des Systèmes Biologiques et des Procédés, CNRS, INRA, INSAT, Université de Toulouse, 31400, France

Supporting information

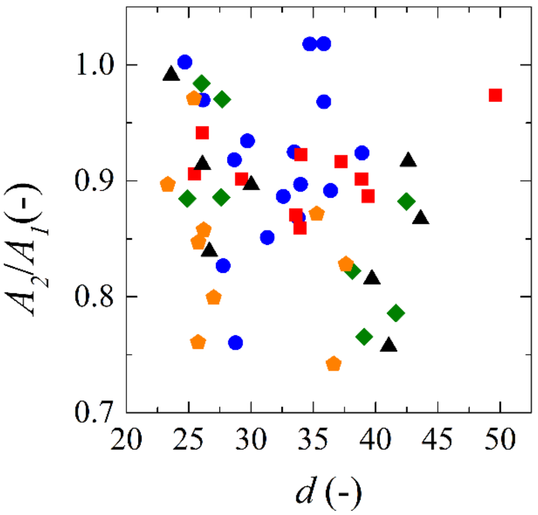

Figure SI. Comparison between deswelling (A2/A1) and pariticle size for constriction angles of: Blue circles 0°, red squares 20°, black triangles 35°, green diamonds 45° and orange pentagons 55°.

- Particle compression

When plotting the area ratio as function of particle size for different entrance angles, we see that microgel deswelling can be as high as 25%, but it is not a function of the particle size or the constriction entrance angle, since the data is very scattered. Small particles may lose up to 25% area, but this is not that much reflected in the circumference, and therefore this parameter can still be close to 1. Also, at high pressure the particles will have less time to deswell and thus need to deform more in order to pass the constriction, or vice versa, and this could be an explanation for range of area ratios that are found.
